# Bcl‐2/Bcl‐xl inhibitor APG‐1252‐M1 is a promising therapeutic strategy for gastric carcinoma

**DOI:** 10.1002/cam4.3090

**Published:** 2020-04-28

**Authors:** Hanjie Yi, Miao‐Zhen Qiu, Luping Yuan, Qiuyun Luo, Wentao Pan, Suna Zhou, Lin Zhang, Xianglei Yan, Da‐Jun Yang

**Affiliations:** ^1^ Department of Experimental Research State Key Laboratory of Oncology in South China Collaborative Innovation Center for Cancer Medicine Sun Yat‐Sen University Cancer Center Guangzhou China; ^2^ Department of Medical Oncology The Second Affiliated Hospital of Nanchang University Nanchang China; ^3^ Department of Medical Oncology State Key Laboratory of Oncology in South China Collaborative Innovation Center for Cancer Medicine Sun Yat‐Sen University Cancer Center Guangzhou China; ^4^ Department of Clinical Laboratory Medicine Sun Yat‐Sen University Cancer Center Guangzhou China

**Keywords:** APG‐1252‐M1, apoptosis, Bcl‐2/Bcl‐xl inhibitor, gastric cancer

## Abstract

Gastric carcinoma is the third major cause of cancer‐related death in China. Bcl‐2 and other BH3 family proteins are critically important in the process of apoptosis pathway, which may be a promising target. APG‐1252‐M1 specifically connects to Bcl‐2 and Bcl‐xl. The antitumor effect of APG‐1252‐M1 in six gastric cancer cells was identified by the Cell Counting Kit‐8 assay. The expression level of proapoptotic proteins was evaluated by Western blot. Meanwhile, the cell cycle and apoptosis distributions were analyzed by flow cytometry and JC‐1. Xenograft models were used to investigate the roles of APG‐1252‐M1 in suppressing the growth of tumors and enhancing the chemotherapy antitumor effect. The antitumor effect of APG‐1252‐M1 was time‐ and dose‐dependent and acted by initiating apoptosis. The change of cell cycle distribution was not discovered in gastric cancer cells treated with APG‐1252‐M1. APG‐1252‐M1 also exhibited synergy with chemotherapy in vivo. The combined group inhibited xenograft tumor growth more obviously than the other groups. Moreover, Ki‐67 was remarkably decreased in the combination group compared to other groups. In conclusion, APG‐1252‐M1 had a strong antitumor effect by inducing apoptosis and was synergistic with chemotherapy.

## Graphical abstract

APG‐1252‐M1 is a new Bcl‐2/Bcl‐xl inhibitor. APG‐1252‐M1 both alone and in combination with chemotherapy exhibited antitumor effects though the intrinsic mitochondrial pathway of apoptosis in gastric cancer.

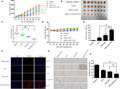

## 1 INTRODUCTION

Gastric carcinoma is the second common malignant tumor in China.^1^ At present, surgery is a radical cure for patients with gastric cancer; but the overall survival of advanced and metastatic gastric cancer is not promising,^2^, ^3^ which has a MST (median survival time) of 10‐12 months.^4^ Therefore, exploring effective new antineoplastic agents in gastric cancer is an urgent clinical need.

Escaping of apoptosis pathway is an important feature of cancer.^5^ Two key features of apoptosis are mitochondrial outer membrane permeabilization and consequent releasing of cytochrome *c*. The Bcl‐2 family proteins regulate the process of mitochondrial outer membrane permeabilization.^6^ The proteins of Bcl‐2 and Bcl‐xl are crucial inhibitors in the procedure of apoptosis and are commonly overexpressed in many tumors;^7^, ^8^ their overexpression is closely related to tumor initiation, aggressive, and chemoresistance.^9^ The resistance of cancer cells to apoptosis mediated by Bcl‐2 is a distinguishing feature of cancer; inhibition of Bcl‐2 antiapoptotic proteins will be a new way to enhance the effectiveness of chemotherapy. Several Bcl‐2 inhibitors have been applied in the treatment of leukemia in recent years.^10^ Our laboratory has reported two Bcl‐2 small molecule inhibitors (ApoG2 and BM‐1197), which had strong antitumor effects in colorectal cancer.^11^ Based on these compounds, our laboratory designed a new agent, APG‐1252‐M1.^12^ Compared to ABT‐263, APG‐1252‐M1 has more effective antitumor activity and fewer hematological toxicities. The mechanism of Bcl‐2/Bcl‐xl inhibitors enhancing chemotherapy is that chemotherapy drugs induce death signaling via activation or inactivation of proapoptotic or antiapoptotic Bcl‐2 family proteins, respectively, and the antitumor effect is amplified by Bcl‐2/Bcl‐xl inhibitors.^13^

In small cell lung cancer xenograft models, APG‐1252 induced complete tumor remission and long‐term tumor regression without serious side effects.^12^ In vivo, APG‐1252 changes to the reactive metabolite named APG‐1252‐M1, which has remarkable antitumor effects in acute myeloid leukemia.^14^

Till now, there is no literature discussing the synergic effect between Bcl‐2 inhibitors and chemotherapy in gastric cancer. APG‐1252 is a novel Bcl‐2/Bcl‐xl inhibitor. In the present study, we aim to prove the antitumor ability of APG‐1252 on gastric cancer and to explore its combination effect with chemotherapy drugs.

## 2 MATERIALS AND METHODS

### 2.1 Reagent and antibody

APG‐1252‐M1 was kindly provided by Ascentage Pharma Group Inc This reagent was dissolved in dimethyl sulfoxide (DMSO; Sigma Aldrich) at a stock concentration of 40 mmol/L, and the final DMSO concentration in the culture media was less than 0.1%. Antibodies against Bcl‐2, Bcl‐XL, Mcl‐1, Caspase 3, Cleaved Caspase 3, BAX, PAPR‐1, and Ki‐67 were purchased from Cell Signaling Technology (CST; USA). Cell culture media and other supplements were obtained from Life Technologies, Inc.

### 2.2 Cell line and cultures

Six cell lines were purchased from the company of Cobioer Biosciences Co. LTD. Before the study, all cell lines were confirmed based on standard cell morphology and were certified by their genomic short tandem repeat (STR) profiles. Cells were cultured in RPMI 1640 medium including 10% fetal bovine serum (Gibco) and 1% streptomycin‐penicillin. All cells were incubated in an environment with 37°C and containing 5% CO_2_.

### 2.3 Cell viability assays

AGS and N87 were seeded at 4 × 10^3^ cells per well in 96‐well plates, and then treated with a series concentrations of APG‐1252‐M1 for 3 days. Cell viability was estimated by cell counting kit‐8 (CCK‐8) (Dojindo) for three times.

### 2.4 Western blot analysis

Cells were cultivated in 6‐well plates with APG‐1252‐M1 or DMSO. After 24‐hours treatment, cells were lysed and resolved on SDS‐PAGE gels and finally transferred to PVDF membranes (Roche). Specific antibodies against Bcl‐2, Bcl‐XL, Mcl‐1, Caspase 3, Cleaved Caspase 3, BAX, PAPR‐1, and GAPDH were added to detect the proteins, and second antibodies were then added to detect primary antibody binding. Bio‐Rad Clarity™ Western ECL substrate was used to detect the antigen‐antibody complexes.

### 2.5 Assessment of apoptosis

AGS and N87 were incubated in a 6‐well cell culture plate (1×10^5^ cells per well) and treated by different concentrations for 2days. After treatment, the cells were then washed by 1x binding buffer, 5μL of Annexin V/FITC was added, and finally incubated for 5minutes. ACEA NovoCyte^TM^ flow cytometry was used to analyze the stained cells. For JC‐1 staining, cells were seeded into 6‐well plates (4×10^3^ cells per well) and cultured for 24hours. The cells were collected and washed with PBS, then incubated at 37°C with JC‐1 for 15minutes. After the dye was washed away, cells were analyzed immediately using flow cytometry (ACEA biosciences).

### 2.6 Analysis of the change of cell cycle

Cells treated with different doses of APG‐1252‐M1 for 48 hours were collected and fixed with 70% ethanol for at least 24 hours. Propidium iodide solution (Beijing 4A Biotech Co. Ltd.) was used to stain DNA. ACEA NovoCyte^TM^ flow cytometry was used to analyze DNA content.

### 2.7 Animal models in vivo

BALB/c athymic nude mice (male, 4 to 6weeks) were provided by the company of Beijing Vital River Laboratory Technology. The mice were raised in the animal facilities of our university. The cells of N87 were mixed with Matrigel at a 1:5 ratio and then subcutaneously injected into the right infra‐axillary of the nude mice. When the tumor volumes arrived 100‐200mm^3^, mice were divided into different APG‐1252 treatment groups (nine mice per group) as follows: control group, 25, 50, and 100mg/kg qd for 10days. In the combined treatment experiments with APG‐1252 and 5‐FU, the mice were separated into four groups (seven mice in each group) as follows: vehicle control group, APG‐1252 group (50mg/kg qd for 10days), 5‐FU (25mg/kg, once a week for 2weeks), and the combined group. APG‐1252 and 5‐FU were given by intravenous injection. At 5weeks after initial implantation when the last tumor volume measurement was obtained, animals were euthanized by carbon dioxide inhalation and followed by cervical dislocation, and subcutaneous tumors were collected and weighed. We recorded tumor sizes and animal weights twice a week, meanwhile tumor volumes were calculated as *V* (mm^3^)=½×(length×width^2^). The animal study complied with the ARRIVE guidelines. The ARRIVE guidelines checklist is shown in Checklist S1.

### 2.8 Immunohistochemical and in situ TUNEL staining

Paraffin sections of xenograft tumor were immunohistochemically stained using Bcl‐2, Bcl‐xl, Mcl‐1, Cleaved Caspase 3, and Ki‐67 antibodies (1:500 dilution), and the stained sections were observed using a Leica microscope. We also used an In Situ Cell Death Detection Kit to stain the animal tissues. The stained slides were imaged and digitized by panoramic MIDI, and the data were analyzed with Panoramic Viewer Software.

### 2.9 Statistical analysis

All the experiment statistical data were analyzed by the software of Prism 5 (GraphPad Software) and expressed as the means ± *SD* (*P < .05; ***P < .001). Meanwhile, all results were conducted with either two‐sided Student's t test or ANOVA test.

## 3 RESULTS

### 3.1 APG‐1252‐M1 restrained the growth of AGS and N87 cells

**Figure 1:**
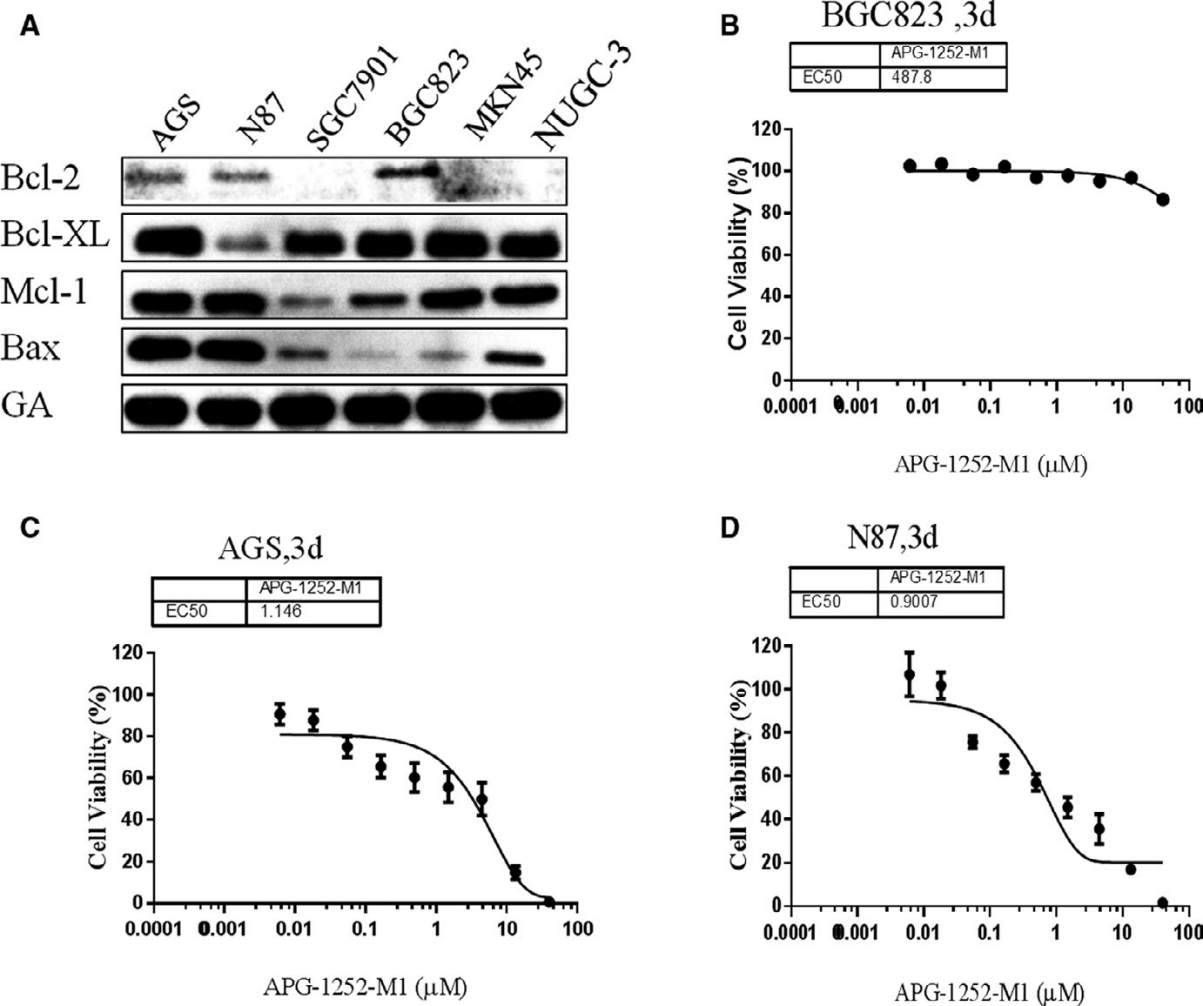
APG‐1252‐M1 inhibited the proliferation of AGS and N87. A, Basal levels of Bcl‐2 family proteins in all six cell lines. B, C, D, Cell viability was detected after treatment with APG‐1252‐M1 for 3 d

We firstly used Western blot to detect the baseline level of Bcl‐2 family proteins in all cell lines without treatment with APG‐1252‐M1 (Figure 1A). Only the AGS and N87 cell lines showed that the expression of Bcl‐2 and Bcl‐xl is concomitant with Bax expression, while the other four gastric cells showed low expression of Bcl‐2 or Bax. Meanwhile, we evaluated the inhibitory ability of APG‐1252‐M1 on gastric cancer cell lines by CCK‐8 assay (Table 1). The results showed that APG‐1252‐M1 inhibited proliferation in cells that expressed Bcl‐2, Bcl‐xl, and Bax (AGS, N87). The IC_50_ values of APG‐1252‐M1 in AGS and N87 cells were 1.146 ± 0.56 μmol/L and 0.9007 ± 0.23 μmol/L, respectively (Figure 1C,D). On the contrary, the IC_50_ values of BGC‐823 with low Bax expression and other gastric cancer cells with low Bcl‐2 expression were all greater than 10 mmol/L (Figure 1B). Therefore, the sensitivity of gastric cancer cell lines to APG‐1252‐M1 was related to the basic level of Bcl‐2, Bcl‐xl, and Bax.

**Table 1:** The IC_50_ of six gastric cancer cell lines to Bcl‐2/Bcl‐xl inhibitor APG‐1252‐M1

Cell lines	IC_50_ (μmol/L)
AGS	1.146 ± 0.56
N87	0.9007 ± 0.23
BGC‐823	487.8 ± 27.6
SGC‐7901	>10 000
MKN45	>10 000
NUGC‐3	>10 000

### 3.2 APG‐1252‐M1 led to caspase 3 activation in AGS and N87 cell lines

**Figure 2:**
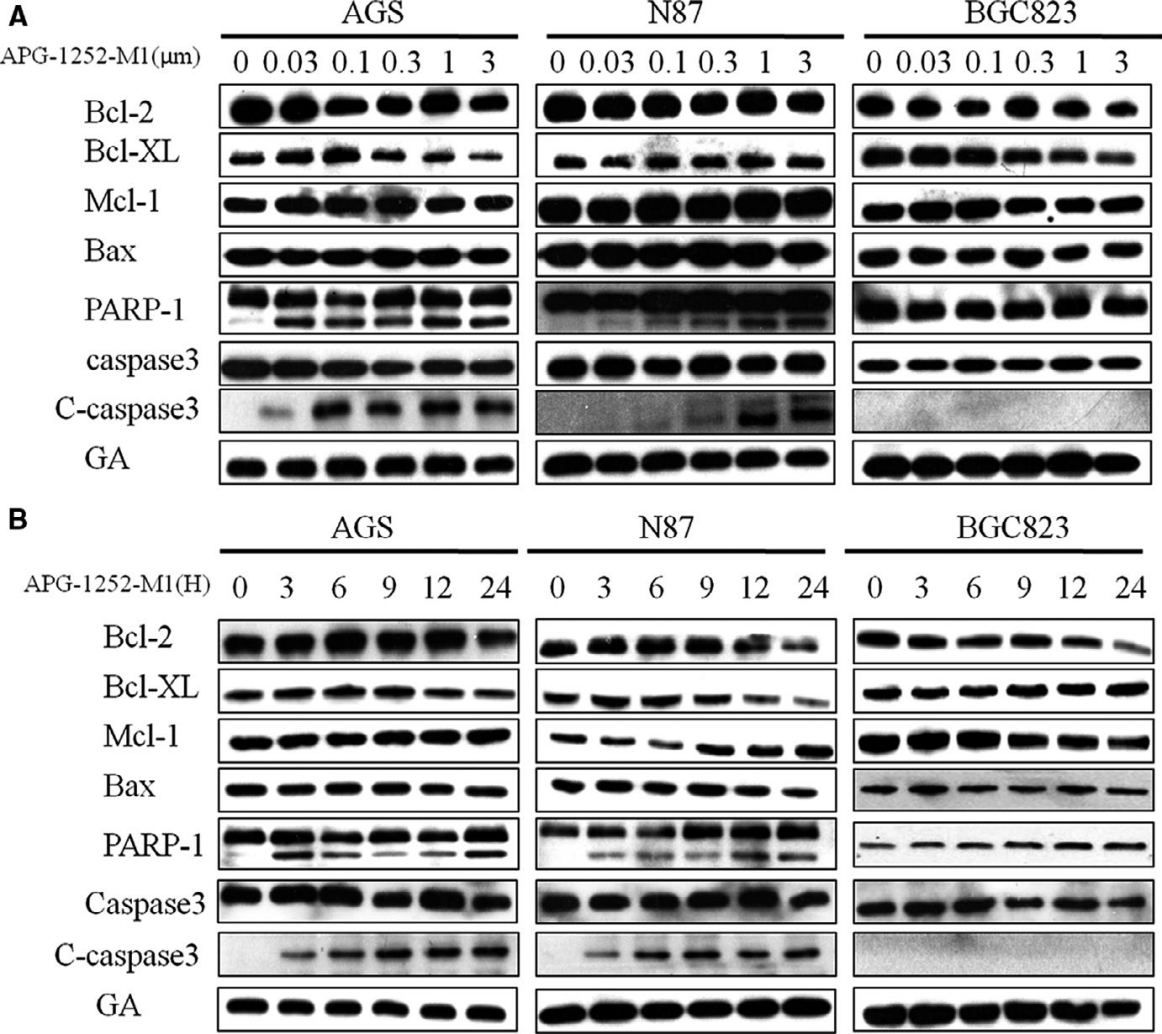
The results of Western blot showed that the level of cleaved PARP and cleaved caspase 3 elevated in AGS and N87 (A, B)

**Figure 3:**
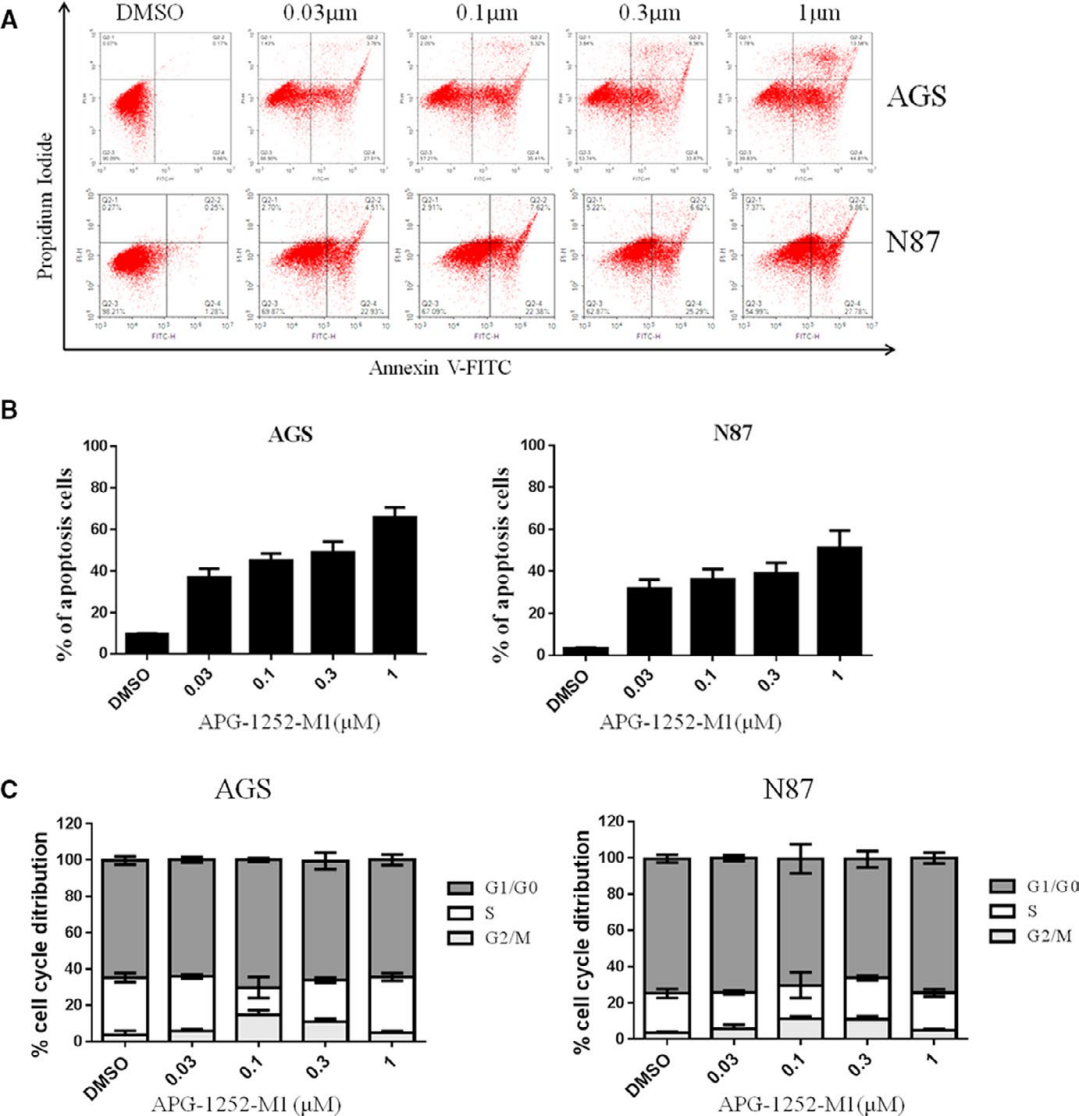
Analysis of apoptosis and cell cycle distribution. A, B, Annexin/FITC staining as assessed showed apoptotic cells in AGS and N87 at 48 h after treatment with different APG‐1252‐M1 concentrations. C, There was no change in the cell cycle distribution of AGS and N87 at 24 h after treatment with different APG‐1252‐M1 concentrations

**Figure 4:**
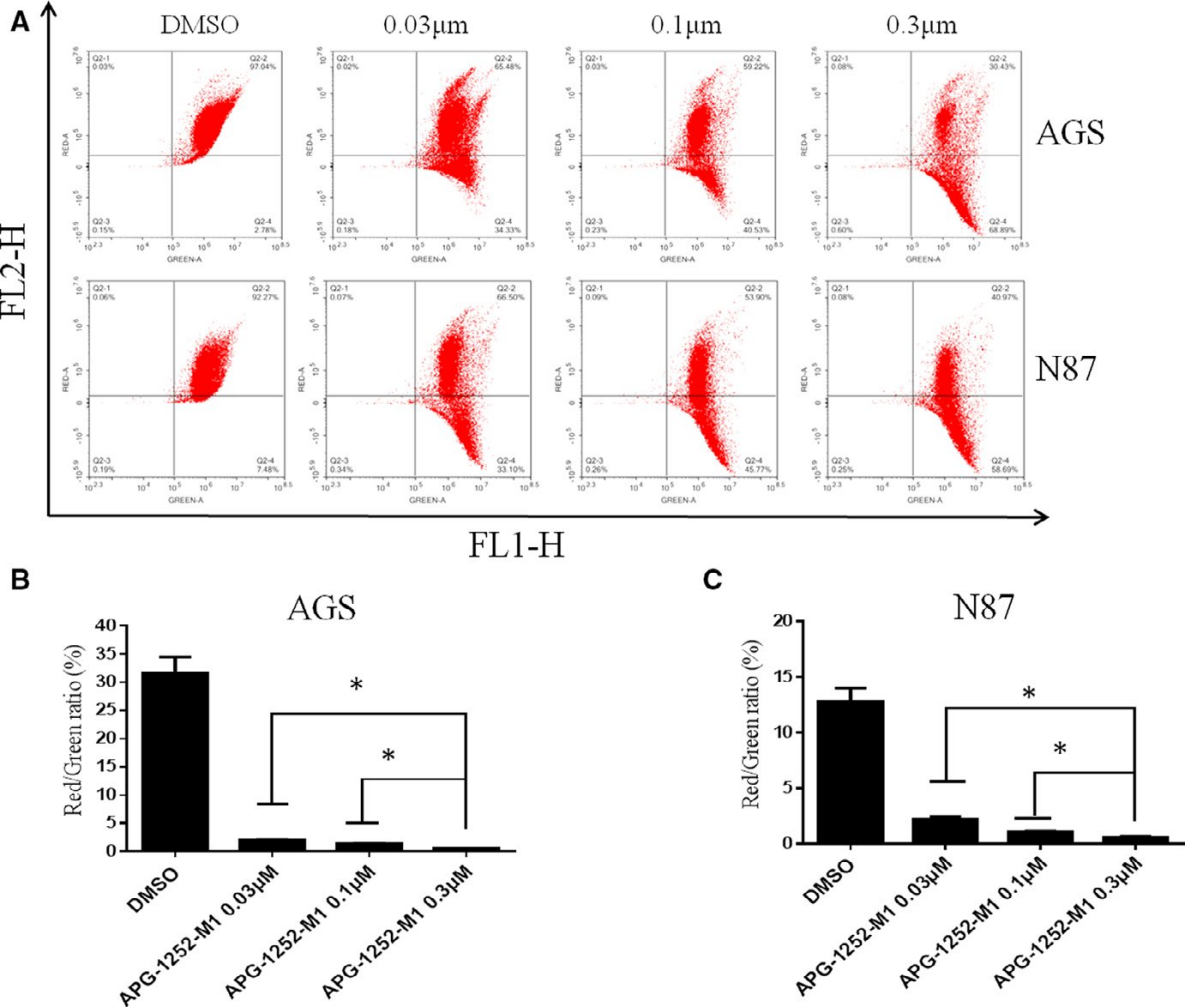
APG‐1252‐M1 induced mitochondrial outer membrane permeabilization (MOMP) in AGS and N87 cells cultured for 24 h before JC‐1 staining. A, The results showed the distribution of JC‐1 aggregates (FLI‐2 channel) and JC‐1 monomers (the FLI‐1 channel) in two cell lines (AGS and N87) with different dose of APG‐1252‐M1. B,C, The ratio of JC‐1 aggregate‐positive and JC‐1 monomer‐positive cancer cells was shown. Experiments were replicated for three times. *P < .05

Western blot analysis showed that the expression of cleaved PARP and cleaved caspase 3 in AGS and N87 cell lines was increased in a concentration‐ and time‐dependent manner with APG‐1252‐M1 treatment (Figure2A,B). The results indicated that inhibiting Bcl‐2/Bcl‐xl could activate the protein of caspase and lead to the release of cytochrome c. The final consequence was apoptosis. We analyzed the distribution between different phase of apoptosis (early and late apoptosis) (Figure3A,B). As the concentration of APG‐1252‐M1 increased, the ratio of apoptotic AGS and N87 cancer cells changed spontaneously; thus, APG‐1252‐M1 had no influence on the cell cycle of either gastric cancer cell lines (Figure3C). The ratio of JC‐1aggregate‐positive and JC‐1 monomer‐positive cells decreased with increasing concentrations of APG‐1252‐M1, which confirmed the antitumor effect of APG‐1252‐M1 via apoptosis (Figure4A‐C).

### 3.3 APG‐1252‐M1 exhibited synergistic activity with a chemotherapeutic drug in vitro experiment

**Figure 5:**
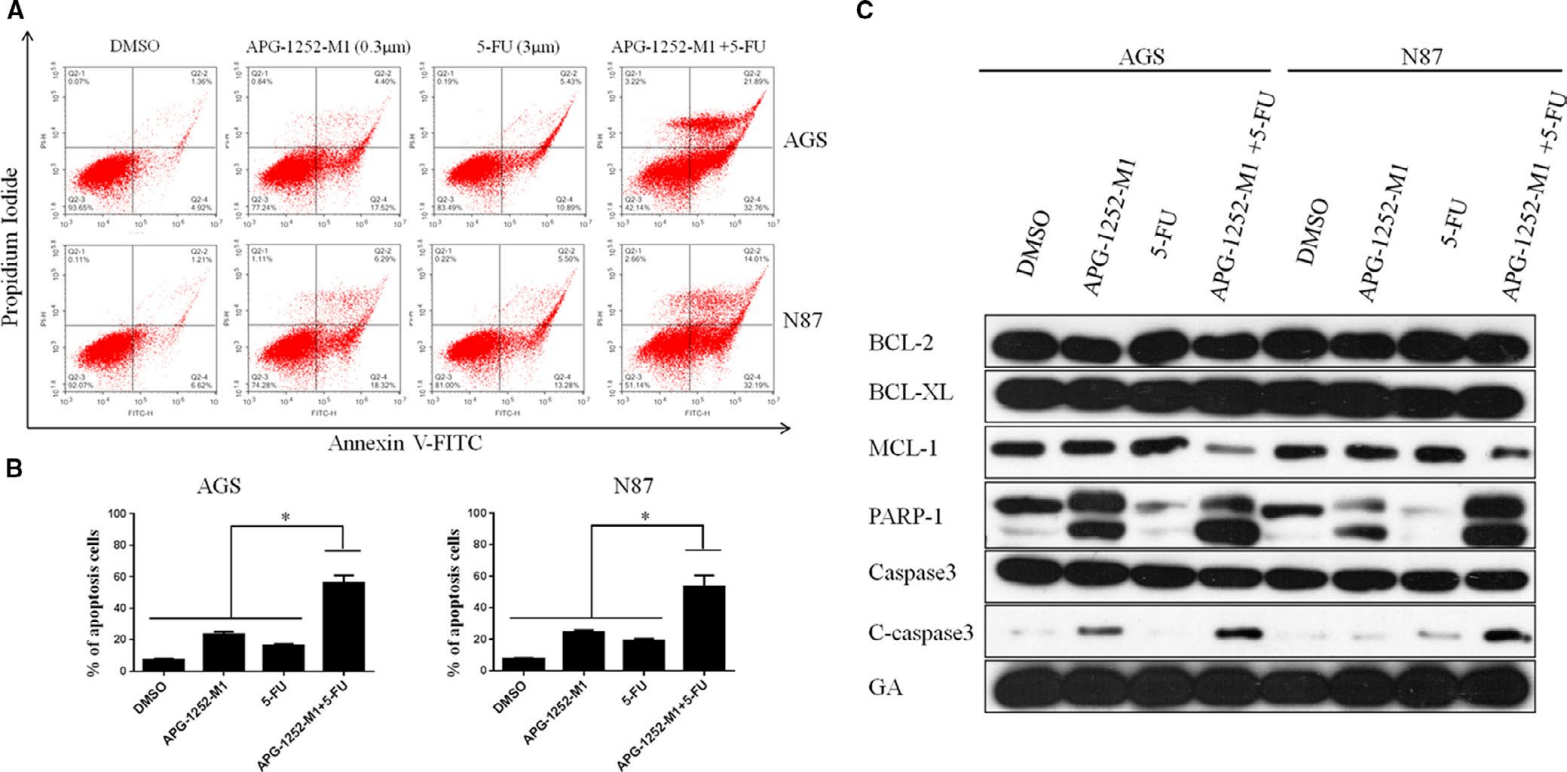
APG‐1252‐M1 exhibited synergy with chemotherapeutic drugs in vitro. Compared with the single treatments and control, the APG‐1252‐M1 plus 5‐FU treatment led to a higher rate of apoptosis in AGS and N87 (A, B; * *P* < .05). (C) Western blot showed that compared to the any single treatment, the combined treatment led to increased expression of the cleaved caspase 3 and PARP‐1 protein in AGS and N87

Annexin V/PI staining by flow cytometry was applied to characterize the apoptosis of cells treated by APG‐1252‐M1, 5‐FU, or both compounds for 48 hours. In AGS cells, treatment with APG‐1252‐M1 or 5‐FU alone resulted in apoptosis rates of approximately 22% and 15%, respectively (Figure 5A,B). In the combination group, the percentage of apoptotic cells was 54%. In N87 cells, the combined treatment group also had more apoptotic cancer cells compared to the single group. Treatment with APG‐1252‐M1 or 5‐FU alone in N87 cells led to apoptosis rates of 24% and 18%, separately (Figure 5A,B), but the combination group caused an apoptosis rate of 46%. The combination treatment in AGS and N87 cells led to increased caspase 3 and PARP‐1 compared to the single treatment groups (Figure 5C). Once again, these data confirmed that APG‐1252‐M1 exhibited synergy with a chemotherapeutic drug in gastric cancer cells.

### 3.4 APG‐1252 suppressed tumor growth in animal model

**Figure 6:**
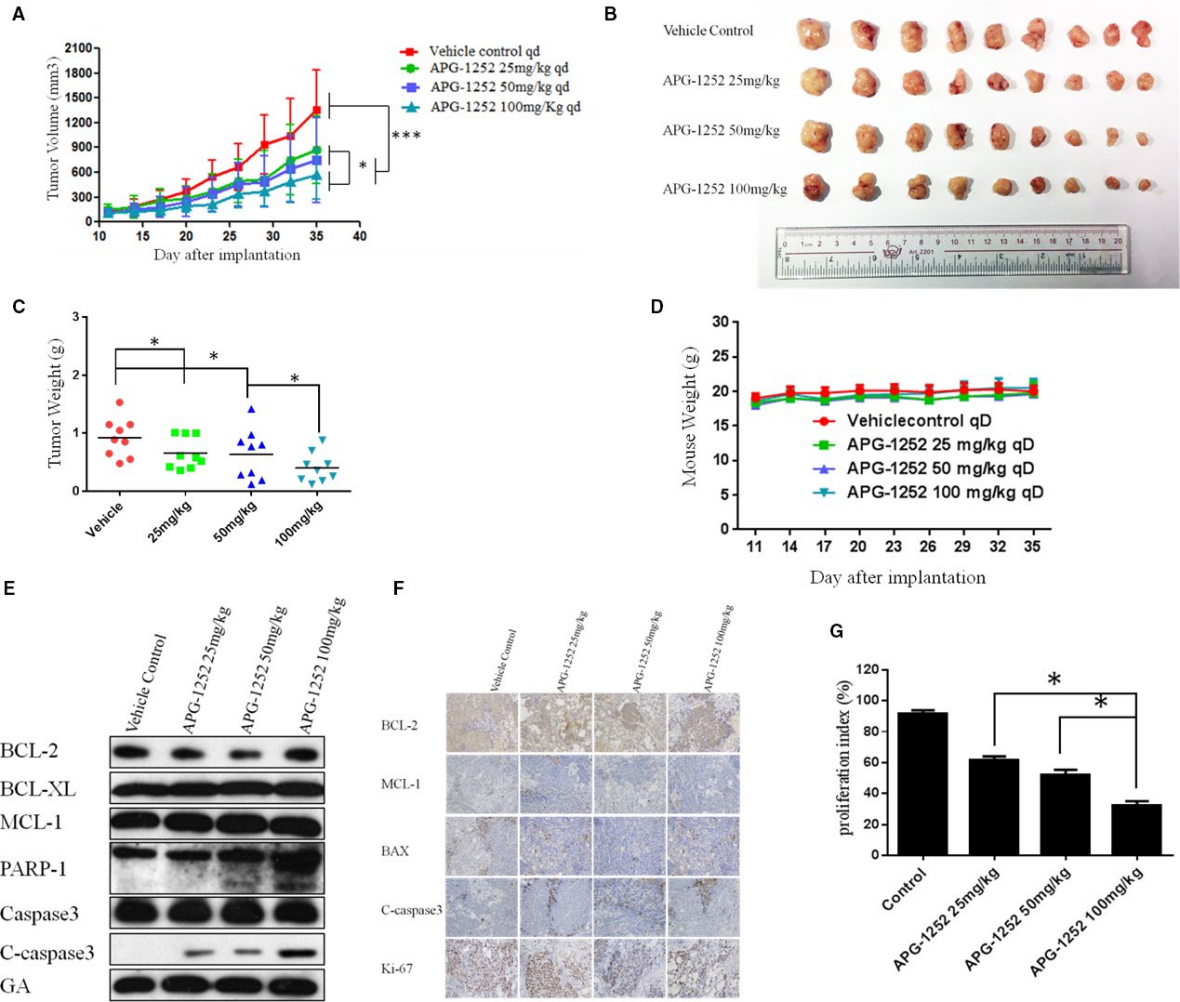
APG‐1252 suppressed tumor growth in vivo. A, Tumor volumes in the four groups. B, Compared to other groups, the tumor volume in the group of APG‐1252 100 mg/kg was the smallest. C, The tumor weight in the group of APG‐1252 100 mg/kg was lightest among the four groups. D, No remarkable diversity in body weight was found among the four groups. E, The results of Western blot show the change of proteins associated with the Bcl‐2 family and DNA damage in tumor tissue of four groups (N87). F, The results of immunohistochemistry show the expression level of BCL‐2, MCL‐1, BAX, Cleaved Caspase 3, and Ki‐67 among four groups. G, Quantification of the Ki‐67 in four groups

To appraise the antitumor effect of APG‐1252, subcutaneously implanted tumor model in nude mouse was administered with different doses of APG‐1252. APG‐1252 alone prohibited the growth of tumors (Figure 6A,B). After 5 weeks, the tumor volumes in the 25 and 50 mg/kg group were, respectively, 700 and 800 mm^3^, whereas the volume in vehicle group reached approximately 1300 mm^3^ (Figure 6A). The tumor volume in the 100 mg/kg group was approximately 400 mm^3^. There were significant differences in four groups. A similar trend was observed in tumor weight (Figure 6C). No differences in body weight were detected in all groups (Figure 6D). Compliance with the data in vitro study, the expression of caspase 3 and PARP‐1 in xenograft tumors gradually increased as the dose increased (Figure 6E). Immunohistochemical staining manifested that the percentage of Ki‐67 in the 100 mg/kg group was distinctly fewer than that in any group (Figure 6F,G).

### 3.5 APG‐1252 synergized with a chemotherapeutic drug in vivo

**Figure 7:**
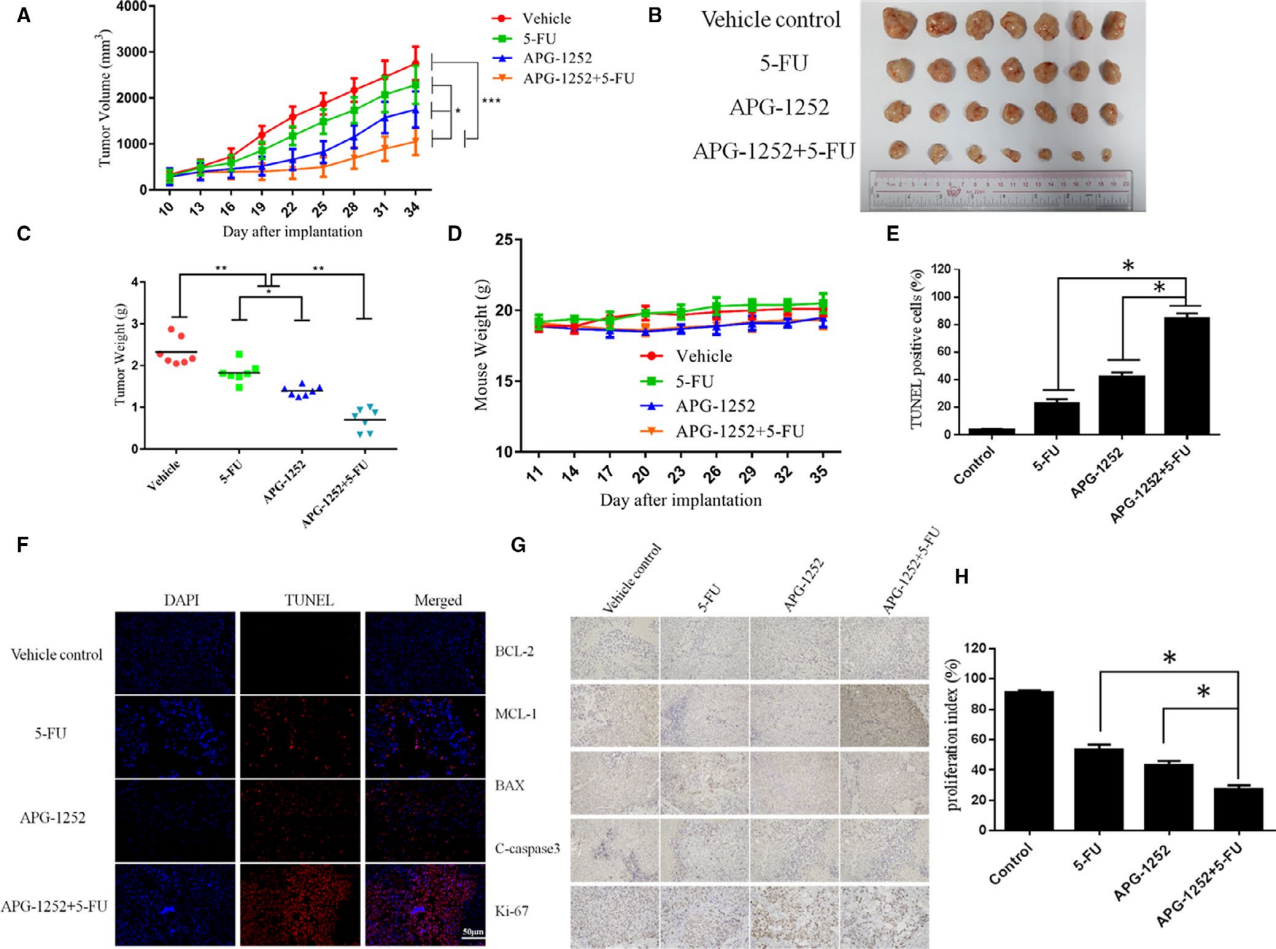
APG‐1252 enhanced the antitumor effects of chemotherapeutic drugs in vivo. A, The xenograft tumor volumes of the combined group grew more slowly than did tumors in the other groups. B, The tumo volume of the combined group was the smallest. C, Compared to the other groups, the tumor weight in the combination treatment group was lightest. D, No significant differences in body weight were found among the four groups. E, F, In situ TUNEL staining showed the extent of apoptosis in a xenograft tumor model. G, We analyzed the level of BCL‐2, MCL‐1, BAX, Cleaved Caspase 3, and Ki‐67 by the method of immunohistochemistry. H, Quantification of the proliferation index (Ki‐67) in the four groups

To investigate the combination of APG‐1252 with chemotherapeutic drugs, nude mice bearing xenograft tumors were divided into four groups: vehicle control, APG‐1252 alone (50 mg/kg), 5‐FU alone (25 mg/kg, once a week for 2 weeks), and both drugs in combination. The tumor volume of the combined group was remarkably smaller than that in the other groups at all measurement times (Figure 7A‐C). No notable differences were found in body weight among the four groups (Figure 7D). The number of TUNEL‐positive cells in the combined group was more than that in the other groups (Figure 7E,F). Compared to any other groups, the combination treatment group showed higher expression of cleaved caspase 3 (Figure 7G). The percentage ratio of Ki‐67 in the combined group was also fewer than that in the APG‐1252 or 5‐FU groups (Figure 7G,H).

## 4 DISCUSSION

Gastric carcinoma is a common malignancy around the world.^15^ Approximately 70% of new cases occur in Eastern Asia, notably in China.^16^ Overexpression of all antiapoptotic proteins restrains the mitochondrial apoptotic pathway, which results in chemoresistance.^17^ APG‐1252 transforms into APG‐1252‐M1 in vivo. Compared with navitoclax (ABT‐263), APG‐1252‐M1 is a nonselective Bcl‐2/Bcl‐xl inhibitor, with high affinity to Bcl‐2/Bcl‐xl, but substantially less affinity to MCL‐1.

In our study, the results of the CCK‐8 assay showed that AGS and N87 were sensitive to APG‐1252‐M1. The mechanism of antitumor activity of APG‐1252‐M1 is consistent with the study as previously reported.^14^ APG‐1252‐M1 induced cytochrome *c* releasing followed by activating the caspase 3 and PARP‐1. Cytochrome *c* activates the caspase signaling pathway and leads to apoptosis.^18^ Two reasons can explain that the cells are sensitive to APG‐1252‐M1 while others are not. First, Bcl‐2 (or Bcl‐xl) is primed with death‐initiating signals. These signals activate monomeric Bax or Bak, which connect with the hydrophobic groove of Bcl‐2 and consequently activate apoptosis.^19^ Second, the initiating death signal must exceed the signaling by Mcl‐1, which is not targeted by APG‐1252‐M1. High levels of Mcl‐1 correlated with resistance to the Bcl‐2 inhibitor in some solid tumors.^20^ Both AGS and N87 not only have high expression of Bcl‐2, but also have the proapoptotic protein Bax, which results in apoptosis; however, SGC‐7901, MKN45, and NUGC‐3 are loss of expression of Bcl‐2, which is the target of APG‐1252. Cell line BGC‐823 has the expression of Bcl‐2 and Bcl‐xl, but without the proapoptotic protein Bax. For these reasons, APG‐1252‐M1 was only effective in AGS and N87 cells, but had no effect on the other four gastric cancer cell lines. We found that AGS and N87 treated with APG‐1252‐M1 underwent apoptosis in a concentration‐ and time‐dependent manner, as shown by the Western blot, JC‐1, and flow cytometry. Moreover, there were no notable changes on cell cycle observed in either cell line. In xenograft animal models, the antitumor activity of APG‐1252‐M1 was increased as the dose increased. However, Bcl‐2 inhibitor alone exerts little efficacy in many solid tumors.^21^ Recent studies have shown that there may be two main reasons. One reason is that some tumors are reliance on antiapoptotic molecules more than Bcl‐2 for survival.^22^ Another reason is that some tumors are dependent on Bcl‐2 to a certain extent and incorporate additional antiapoptotic molecules.^23^ Therefore, combining a Bcl‐2 inhibitor with chemotherapy may be an effective way to inhibit the growth of tumors. Most chemotherapeutic drugs initiate the intrinsic signal pathway of apoptosis to kill tumor cells, but it is easy for tumor cells with high level of Bcl‐2 to evade apoptotic signal pathway. Therefore, there is a need to provide additional support to combat chemotherapy resistance.^24^ Many studies have confirmed that BH3 mimetics enhanced the apoptotic response in various cancers when combined with traditional chemotherapy drugs.^25^ Therefore, chemotherapeutic drugs may provide an additional important event required to empower the Bcl‐2 inhibitor to eliminate resistant cancer cells. Our study showed that APG‐1252‐M1 synergized with chemotherapeutic drugs not only in vitro but also in xenograft animal models. The combination treatment group induced more apoptosis than any of the single treatment groups in vivo and in vitro.

However, there are some limitations worth noting in this study. First, we found that only AGS and N87 were sensitive to APG‐1252. The gastric cancer cell line of AGS was unable to form transplant tumor in nude mice. Therefore, we chose N87 to establish the xenografts in nude mice, which is a HER2‐positive gastric cancer cell line. It does not represent all gastric cancers. Therefore, it is necessary to expand the number of cancer cell lines to further clarify the antitumor effect of APG‐1252‐M1 in gastric cancer. Second, only APG‐1252‐M1 plus 5‐FU was used for the combination treatment. The next step is to combine APG‐1252‐M1 with other common chemotherapy drugs, such as cisplatin and paclitaxel, for treating gastric cancer. Finally, additional toxicities (such as hematological toxicity) for single‐agent and combination therapies require further analysis.

In summary, our study indicated that APG‐1252‐M1 both alone and in combination with chemotherapy exhibited antitumor effects though intrinsic mitochondrial pathway of apoptosis in gastric cancer.

## COMPETING INTERESTS

Not applicable.

## ETHICS APPROVAL AND CONSENT TO PARTICIPATE

Sun Yat‐Sen University Committee for Use and Care of Laboratory Animals approved the study.

## AUTHOR CONTRIBUTIONS

YHJ, YXL, and QMZ carried out the main work and drafted the manuscript. YLP and ZSN carried out the Western blot and immunohistochemistry. LQY and PWT carried out the in vivo study. ZL performed the statistical analysis. QMZ and YDJ designed the study. All authors read and approved the final manuscript.

## CONSENT FOR PUBLICATION

The manuscript have been read and approved by all authors.
